# Treatment of symptomatic macromastia in a breast unit

**DOI:** 10.1186/1477-7819-8-93

**Published:** 2010-11-01

**Authors:** Fernando Hernanz, Rosa Santos, Arantxa Arruabarrena, José Schneider, Manuel Gómez Fleitas

**Affiliations:** 1 Department of Surgery, University of Cantabria, Hospital "Marqués de Valdecilla", Avda Valdecilla s/n, 39008 Santander, Cantabria, Spain; 2 Department of Gynecology, University of Cantabria, Hospital "Marqués de Valdecilla", Avda Valdecilla s/n, 39008 Santander, Cantabria, Spain; 3 Breast Cancer Unit, University of Cantabria, Hospital "Marqués de Valdecilla", Avda Valdecilla s/n, 39008 Santander, Cantabria, Spain

## Abstract

**Background:**

Patients suffering from symptomatic macromastia are usually underserved, as they have to put up with very long waiting lists and are usually selected under restrictive criteria. The Oncoplastic Breast Surgery subspeciality requires a cross-specialty training, which is difficult, in particular, for trainees who have a background in general surgery, and not easily available. The introduction of reduction mammaplasty into a Breast Cancer Unit as treatment for symptomatic macromastia could have a synergic effect, making the scarce therapeutic offer at present available to these patients, who are usually treated in Plastic Departments, somewhat larger, and accelerating the uptake of oncoplastic training as a whole and, specifically, the oncoplastic breast conserving procedures based on the reduction mammaplasty techniques such as displacement conservative techniques and onco-therapeutic mammaplasty. This is a retrospective study analyzing the outcome of reduction mammaplasty for symptomatic macromastia in our Breast Cancer Unit.

**Methods:**

A cohort study of 56 patients who underwent bilateral reduction mammaplasty at our Breast Unit between 2005 and 2009 were evaluated; morbidity and patient satisfaction were considered as end points. Data were collected by reviewing medical records and interviewing patients.

**Results:**

Eight patients (14.28%) presented complications in the early postoperative period, two of them being reoperated on. The physical symptoms disappeared or significantly improved in 88% of patients and the degree of satisfaction with the care process and with the overall outcome were really high.

**Conclusion:**

Our experience of the introduction of reduction mammaplasty in our Breast Cancer Unit has given good results, enabling us to learn the use of different reduction mammaplasty techniques using several pedicles which made it posssible to perform oncoplastic breast conserving surgery. In our opinion, this management policy could bring clear advantages both to patients (large-breasted and those with a breast cancer) and surgeons.

## Background

Oncoplastic breast surgery (OBS), understood as the seamless joining of the extirpative and reconstructive aspects of breast surgery that is performed by a single surgeon, is an efficient model which requires a new specialized training [1]. In our opinion, mammaplasty techniques are a very important skill which makes it possible to perfom a variety of options in the context of OBS: onco-therapeutic mammaplasty, volume displacement oncoplastic procedures, controlateral symmetry procedures relative to the opposite breast in breast reconstruction or surgical correction of cosmetic sequelae after breast conserving surgery [2].

We have adopted this model of OBS, comprehensive breast surgeon who performs oncologic and reconstructive procedures, and reduction mammaplasty (RM) has been included in the service catalogue of our Breast Cancer Unit (BCU) in an attempt to achieve two main objetives:

a) to increase the offer of treatment to patients with symptomatic macromastia who are an underserved population having to put up with a long waiting list and b) to make the uptake of the new oncoplastic training easier and quicker because one of the disavantages of this oncoplastic model is that not only is the training programme long but also not commonly available.

The aim of this retrospective study is to analyze the outcome of RM for symptomatic macromastia in our BCU and comment upon two experiences using different types of mammaplasty in the context of OBS.

## Methods

A cohort of 56 patients suffering symptomatic macromastia, all of them satisfying at least one of the selection criteria: distance from the nipple to sternal notch longer than 33 cm, gigantomastia (the amount of breast tissue needed to be resected bigger than 1000 g per breast), specialist recommendation justified in traumatological or psychological problems, underwent bilateral RM at our BCU between 2005 and 2009. Demographic and perioperative data were collected (Table 1). Regardless of the type of pedicle used to lift the nipple areola complex (NAC) the perioperative management of these patients consists of certain common measures.

**Table 1:** Patient characteristics and operative data.

Number of Patients	56
**Age, years**	
Mean	42
Range	29-67

**Risk Factors, (percentage)**	
**Smoking habit**	
Yes	9 (16)
No	47 (84)
**Co-Morbidity (Diabetes mellitus, arterial hypertension)**	
Yes	7 (12.5)
No	49 (87.5)
**Body-mass index, Kg/m^2^**	
Mean	30.56
Range	21-43.5

**N-SN distance, cm**	
Mean	33.85
Range	26-45

**Nipple elevation, cm**	
Mean	7
Range	7-19

**Weight of gland resected per breast, g**	
Mean	652.50
Range	95-2020

**Type of pedicle (percentage)**	
Superomedial	9 (17)
Inferior	3 (5.3)
Vertical bi-pedicle	41 (73)
Free nipple graft	3 (5.3)

Abreviation: N-SN, nipple-to-sternal notch

Smokers were strongly urged to give the habit up, and if they did not do so, they were clearly informed of the high risk of serious complications before being operated on. Patients were fully informed, and were required to sign a specific informed consent form. This form includes some sketches, information on visible scar locations, text describing the main complications and where our general surgery specialty is explicity expressed; there also appear some photographs showing the average cosmetic outcome.

A mammogram before breast reduction is not obligatory, the main reason being that the waiting list is so long that mammograms will be unuseable if they were to be done at the time the patient is included on the list and then once surgery is planned there is only a short time available. Patients aged 50 and over had frequently had a screening mammogram carried out by the government programme for breast cancer detection within the preceding two years.

All surgical procedures were carried out under general anesthesia by a breast surgeon (Hernanz, F) and on an inpatients basis, the hospital stay was very short, one-two days.

All patients had antibiotic (one preoperative intravenous dose of cephalosporine) and deep venous thrombosis prophylaxis, stocking and chemoprophylaxis being administered subcutaneously. No tumescent solution infiltration was used.

We used light suction drainages in all patients, two per breast, which were placed through the incisions and fixed with adhesive, so that they could be taken out by pulling down on them, usually on the second postoperative day, without the dressing bandage having to be removed or released. Breast incisions were topped with sterile adhesive plaster in the operating room, and these were removed in the clinic a week later. A soft bandage was put on, except for the few cases with NAC free graft, at the top of which a window was made to monitor NAC viability and to enable patients to carry out a circular massage every hour during the early postoperative days thus avoiding venous congestion of NAC. In the first clinic visit, a week after surgery, the bandage was removed and a nonwired support bra was put on, this having to be worn until the end of the second month. Intradermical sutures were taken out at the third week.

All breast reduction specimens were submitted for pathological assessment. Three months after surgery a mammogram was taken to serve as a baseline study with which to compare further studies.

Morbidity and patient satisfaction were evaluated as our endpoints.

Data were collected by reviewing medical records, and then, at least six months after surgery, 47 patients willing to be interviewed were interviewed by one of the authors (Santos, R). The inteview contains nine questions which are related with six subject areas: satisfaction with the breast, satisfaction with overall outcome, psychosocial well-being, sexual well-being, physical well-being and satisfaction with the care process, these areas being considered the main issues of concern for breast surgery [3].

## Results

Eight patients (14.2%) presented complications in the early postoperative period, two of them being reoperated on for evacuation of a hematoma and an abcess. The remaining complications were: hematoma (3), T-junction dehiscence (2), necrosis of the skin flaps (1). None had a total or partial necrosis of NAC. Thirty patients (64%) of those interviewed presented some change in nipple sensation, with a reduction of sensation in 16 (34%) and absence in 7 (14.8%). In the late postoperative period, four patients were diagnosed via mammograms as having a focus of fat necrosis and one epidermic cyst which was extirpated by local anesthesia. The result of the satisfaction survey is shown in Table 2.

**Table 2:** Satisfaction survey: data from 47 patients interviewed (themes and queries)

**Satisfaction with breast**.	Number
Are you satisfied with the breast size?	
No	2
only a little	1
fairly	5
quite	20
yes	19
	
Are you satisfied with the appearance of the scars?	
Yes	41
No	6
	
Considering 1 as very bad and 10 as excellent, how do you score the overall cosmetic outcome?	
1-4 (bad)	3
5-6 (fair)	8
7-8 (good)	11
9-10 (excellent)	25

**Satisfaction with overall outcome**	
Would you recommend it to anybody who is thinking about it?	
Yes	100
No	0

**Psychosocial well-being**	
Have the psychological symptoms which you have been suffering from disappeared?	
No	20
only a little	1
fairly	2
quite a lot	10
yes	14

**Sexual well-being**	
Have your sexual relations improved?	
Yes	14
No	33

**Physical well-being**	
Have the physical symptoms which you have been suffering from disappeared?	
No	2
only a little	1
fairly	2
quite a lot	13
yes	29

**Satisfaction with the care process**	
Do you consider that you have received sufficiently complete preoperative information about the surgical process?	
Yes	36
No	11
	
Are you satisfied with the care provided?	
Yes	46
No	1

## Discussion

Previous experiences in RM performed by general surgeons reported similar outcomes to plastic ones, the purposes that motivated these practices in the nineties being to provide surgical care to an underserved population and to increase the 'general surgeon's' range of skills [4-6]. These motives are very much in vogue at the moment, and what is more, they have been strengthened by the appearance of OBS.

Although RM has proved to be efficacious in reducing the symptoms and in improving the quality of life for patients with macromastia, and despite the effect of RM being comparable to other unquestionable surgical procedures such as hip and knee total joint replacement [7], the fact is that, in the private health sector, this procedure is only covered by the insurance companies with very restrictive conditions (most insurance carriers do not reimburse for this surgery when it involves less than a specific amount of breast tissue being resected), whereas, in our public health system, in which the conditions are less rigorous, the waiting lists are very long; it is clear, then, patients with symptomatic macromastia are underserved.

Over the last decade OBS has gradually spread all over the world [1] and has just been considered as the gold standard for breast conserving surgery[8]; however, regardless of the oncoplastic model chosen (comprehensive breast surgeon or oncoplastic team), oncoplastic training is needed. Because of the barriers between specialities this cross-speciality training is difficult and is not easily accessible with the exception of the United Kingdom where an oncoplastic fellowship was created in 2002 [9].

The introduction of RM in the catalogue of a BCU for the treatment of patients with symptomatic macromastia (usually treated in Plastic Departments) could have a sinergic effect. By treating these patients in a BCU an underserved population is provided with a larger offer and the uptake of oncoplastic training, which in the end means an improvement in the quality of breast cancer surgery, is facilitated. Patients, whether large-breasted or with breast cancer, and surgeons both clearly benefit from this management organization.

We have introduced RM in our BCU with criteria for inclusion on the waiting list that must be satisfied. Two of these criteria, which are related with the nipple to sternal notch distance (> 33 cm) or with the weight of breast tissue which has to be removed (> 1000 g per breast), are arbitrary limits that do not take into consideration either a patient's height or weight or their symptoms or the deterioration in quality of life. We are fully aware that some patients who did not satisfy any of our selection criteria could clearly benefit from a RM.

36 (64.28%) patients were included in our waiting list complying the criteria related with the distance from the nipple to sternal notch being the most frequent criteria. In 10 patients the amount of breast tissue excised was equal or bigger than 1000 g but the patients included for this criteria were 15, the reason for this different it is that this criteria is an preoperative estimation based on the surgeon experience and it could be inaccurate; our experience with mathematical models which calculate this amount using several variables as IMC, distance from the nipple to the infra-mammary fold, etc, is that they overestimate it. Other 12 (20%) patients were included with inform from a specialist (orthopedic, rheumatologist, physiotherapist) who recommends the reduction mammaplasty as way to improve a concomitant pathology.

According to our results, the majority of patients were satisfied with the cosmetic outcome and their final breast size, only 3 patients considering the cosmetic results as bad and another 3 patients wishing the surgeon had carried out a larger resection. As might well be expected, the physical symptoms disappeared or significantly lessened in 88% of patients because our selection criteria imply that all selected patients had a symptomatic macromastia and in 34 of interviewed patients (72.34%) back pain was the main reason for being operated on.

Although the degree of satisfaction with the care process and with the overall outcome was high we are concerned about data showing that 23% of patients felt that they had not received appropriate information about the surgical procedure. In this type of surgery, we consider that information is an essential part of the overall process, so patients must be fully informed about the surgical procedure and its potential complications, which could be cause of serious cosmetic sequelae such as loss of the nipple areola. Taking in account this data the information process will be improved and we think it would be a good idea to arrange a visit the week before the surgery to focus on this point. At the same time a mammogram could be taken in women of 40 or over, providing us with a good opportunity to detect occult carcinomas because mammograms taken within one year preceding surgery are not an accurate detection test [10]. We found neither occult carcinomas nor any significant pathologic findings in the breast tissue removed, although our series has an average age of 42 years and pathologic findings increase significantly in patients over 40.

We have used a variety of pedicles with the same incision pattern (T-inverted), the pedicle chosen depending on several variables such as projected nipple movement, risk of serious complications and patient/surgeon preferences; we have also taken into account the use of different technical options thinking wherever possible of their application in OCS because real versatility is needed to cope with the different situations that could arise [11-13].

**Figure 1:**
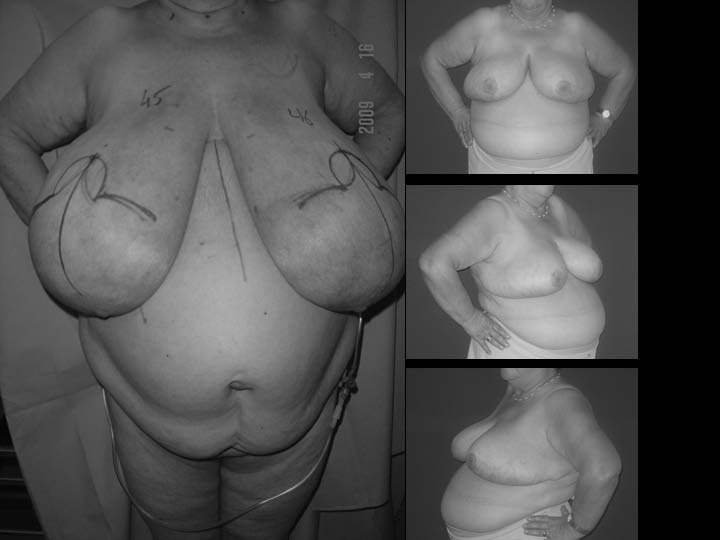
**Patient with severe symptomatic macromastia**. A 67-year-old woman with gigantomastia, who was treated using an RM with free nipple areola graft because she had several complication risk factors such as BMI 39, arterial hipertension, diabetes and projected movement of the NAC longer than 15 cm and, in addition, she was not worried about nipple conservation. The amount of breast tissue resected weighed 3626 g. Appearance before and five months after breast reduction.

**Figure 2:**
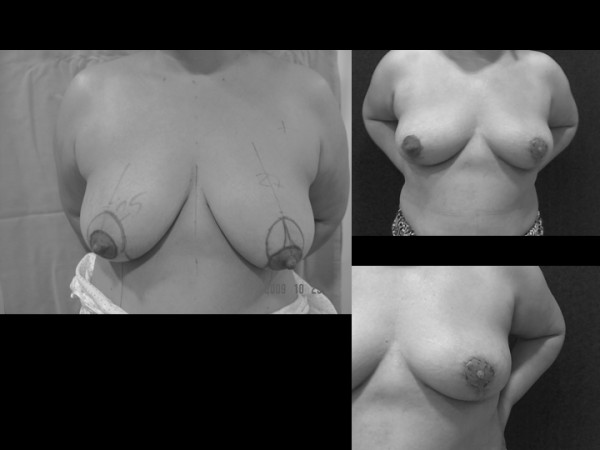
**Oncoplastic breast conserving surgery. Central tumor treated using a mammaplasty technique**. A 52-year-old woman who presented with an invasive ductal carcinoma situated in the retro-areolar area of the left breast with a complete response after neoadjuvant chemotherapy was treated by oncoplastic conserving surgery using an onco-therapeutic mammaplasty (central cuadrantectomy and reshaping). Below left. Nipple areola complex reconstructed using a free graft from the skin of the right breast. Appearance before and one month after surgery.

Free nipple areola graft has been used in only three patients, who have a high risk of complications (high Body Mass Index (BMI), comorbidity and big resection is needed) and they had no interest in nipple sensitivity or breast feeding preservation (Figure 1). We think that knowledge and management of this technique is very useful in OBS for central quadrant tumors involving the nipple (Figure 2).

The inferior pedicle is easier and safer and in OBS can be used in tumors situated in all other quadrants of the breast [14], but as time passes it is frequently accompanied by pseudoptosis or bottoming.

Although excellent results can be produced with a variety of procedures the latest patients have been preferably treated using a superomedial pedicle to transport NAC and the inferior one used to make an inferior cone pole to achieve an appropriate breast projection; the good cosmetic outcomes and the safety and reliability for big resections [15,16] are two important reasons which have led to this technique becoming our first choice.

**Figure 3:**
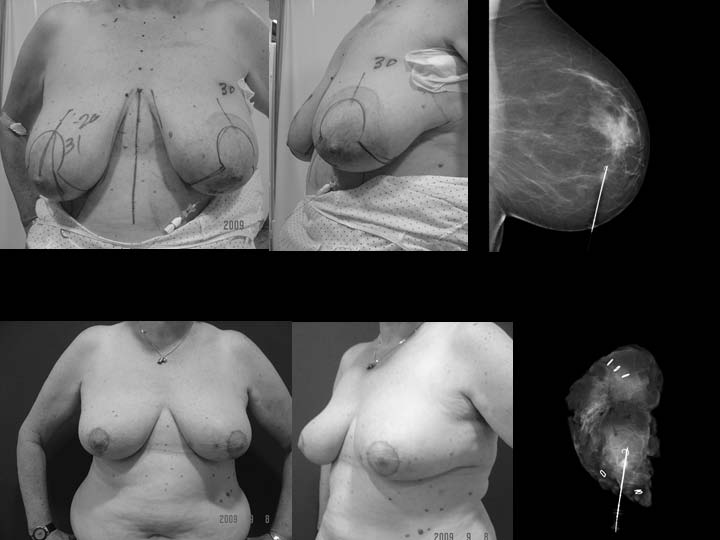
**Oncoplastic breast conserving surgery. Breast cancer and macromastia treated using a mammaplasty technique**. A 58-year-old woman with large breasts who presented with an invasive small ductal carcinoma of 7 mm. in the inferior retroareolar area of the left breast diagnosed by screening programme. She was treated using an onco-therapeutic mammaplasty with a T-inverted pattern incision and a superomedial pedicle to transpose the NAC to 6 cm up and the inferior one to increase the inferior pole breast projection. Above. Preoperative view. We used a wire for tumor location. Sentinel lymph node biopsy was carried out resulting negative. On the left side, above, mammogram with a wire inserted in the tumor. On the left side, below, the x-ray test of the surgical specimen of a really wide resection weighing 175 g. can be seen. Below. Appearance at five weeks postoperatively with a good cosmetic outcome before adjuvant radiotherapy. It can be seen that there are shoulder bra strap groovings and that the left breast is intentionally slightly bigger than the right one because the effect of radiotheraphy would equalize them.

Another reason for this statement is that the superomedial pedicle is used in the management of tumors situated in the upper outer quadrant, the most frequent location. It can also be used in other tumor locations such as in the patient shown in Figure 3, who had a small tumor located in the retro-inferior-areolar area of a large left breast. By using an onco-therapectic mammaplasty (Wise pattern incision with superomedial pedicle for traslating the NAC to an inferior one to improve the breast projection) the patient enjoyed certain advantages [17]: a wide tumor resection avoiding any cosmetic sequelae, a reduction in the size of her breasts offering a medium size left breast to the radiotherapist and relieving such symptoms as neck pain and, finally, an improvement in her body-image thanks to the good cosmetic outcome.

Nevertheless, the possible disadvantages that this management policy could bring to the BCU should also be discussed. The main one it is that RM is a time-consuming procedure. In this series of patients the average operating time was 200 minutes but what should be taken in account is that, to begin with, it takes longer and the operating time varies much more because it all depends on surgical skill level [18], so the majority of cases are performed as an isolated procedure; for that reason our offer is limited to about 20 patients per year. This number might be enough to improve oncoplastic training but it is clearly insufficient for the demand from large-breasted patients.

## Conclusion

Our experience of introducing RM in our BCU has given good results with low morbidity and a high degree of patient satisfaction. In our opinion, this synergic management policy increases the scarce therapeutic offer available to these patients and has led to a faster uptake of oncoplastic training, bringing clear advantages both to patients (larged-breasted patients and patients with breast cancer) and surgeons.

## Abbreviations

(OBS): Oncoplastic breast surgery; (RM): Reduction mammaplasty; (BCU): Breast Cancer Unit; (NAC): Nipple areola complex; (BMI): Body Mass Index.

## Competing interests

The authors declare that they have no competing interests.

## Authors' contributions

HF, general surgeon who carried out the surgical procedures and principal investigator, participated in design and coordination of the study.

SR, gynaecologist resident who participated in data collecting and conducted the patient interviews.

AA, resident general surgeon who participated in data collecting and surgical procedures

SJ, chief of Breast Cancer Unit participated by reviewing the article.

GFM, chief of Surgical Department participated by reviewing the article.

All authors read and approved the final manuscript.

## Consent

Written informed consent was obtained from the patient for publication of this case report and accompanying images. A copy of the written consent is available for review by the Editor-in-Chief of this journal.
